# Aminoacids and Flavonoids Profiling in Tempranillo Berries Can Be Modulated by the Arbuscular Mycorrhizal Fungi

**DOI:** 10.3390/plants8100400

**Published:** 2019-10-08

**Authors:** Nazareth Torres, Ghislaine Hilbert, María Carmen Antolín, Nieves Goicoechea

**Affiliations:** 1 Plant Stress Physiology Group, Department of Environmental Biology, Schools of Sciences and Pharmacy and Nutrition, Universidad de Navarra, Associated to CSIC (EEAD, Zaragoza, ICVV, Logroño), 31008 Pamplona, Spain; ntorres@alumni.unav.es (N.T.); cantolin@unav.es (M.C.A.); 2 EGFV, Bordeaux Sciences Agro, INRA, Université de Bordeaux, 33883 Villenave d’Ornon, France; ghislaine.hilbert@inra.fr

**Keywords:** aromatic precursors, berry skin metabolites, mycorrhizal symbiosis, phenolic compounds, soluble sugars, *Vitis vinifera*

## Abstract

(1) Background: *Vitis vinifera* L. cv. Tempranillo is cultivated over the world for its wine of high quality. The association of Tempranillo with arbuscular mycorrhizal fungi (AMF) induced the accumulation of phenolics and carotenoids in leaves, affected the metabolism of abscisic acid (ABA) during berry ripening, and modulated some characteristics and quality aspects of grapes. The objective of this study was to elucidate if AMF influenced the profiles and the content of primary and secondary metabolites determinants for berry quality in Tempranillo. (2) Methods: Fruit-bearing cuttings inoculated with AMF or uninoculated were cultivated under controlled conditions. (3) Results: Mycorrhizal symbiosis modified the profile of metabolites in Tempranillo berries, especially those of the primary compounds. The levels of glucose and amino acids clearly increased in berries of mycorrhized Tempranillo grapevines, including those of the aromatic precursor amino acids. However, mycorrhizal inoculation barely influenced the total amount and the profiles of anthocyanins and flavonols in berries. (4) Conclusions: Mycorrhizal inoculation of Tempranillo grapevines may be an alternative to the exogenous application of nitrogen compounds in order to enhance the contents of amino acids in grapes, which may affect the aromatic characteristics of wines.

## 1. Introduction

The symbiotic association of plants with arbuscular mycorrhizal fungi (AMF) is a common phenomenon observed in nearly 80% of plant species, including grapevines [1,2]. The composition of the AMF communities in vineyards around the world is dominated by genus and species belonging to the family Glomeraceae [3]. The association of *Vitis vinifera* L. with AMF can enhance the uptake of mineral nutrients from the soil, thus benefiting plant vigor [4], and improves the tolerance of grapevines against abiotic [5] and biotic [6] stresses. The influence of AMF on plant metabolism is not restricted to the target organ (root), but it can also affect the physiology and metabolism of the aerial part, including fruits [7]. There is an increasing number of studies focused on the effect that AMF can exert on the quality of plant edible parts [8]. In regard to grapevines, Torres et al. [3] defined AMF as a promising resource for improving berry quality in grapevines in changing environments.

Tempranillo grapevine (also known as Tinta Roriz, Aragonez or Valdepeñas) is widely cultivated in several countries over the world (Argentina, Australia, France, Portugal, Spain, and the USA) for its wine of high quality. This variety of grapevine accounts for the 21% of the total Spanish vineyard surface [9], with Spain being the leader country in 2017 in terms of cultivated surface area (967 kHa) [10]. Research performed under greenhouse controlled conditions demonstrated that AMF can induce the accumulation of phenolic compounds and carotenoids in leaves of Tempranillo grapevines, although results may vary depending on the high intravarietal diversity found in this variety [11]. In contrast, environmental factors, such as air temperature and water irrigation regime, were more decisive than mycorrhizal symbiosis for berry quality [12,13]. However, under conditions that simulated future climate change scenarios (elevated temperatures and drought, acting alone or in combination), the application of mycorrhizal inoculum modulated some concrete aspects related to characteristics (berry mass, relative skin mass) and quality (must pH, total anthocyanins, total antioxidant capacity, and concentration of malic and tartaric acids) of grapes [12,13]. Mycorrhizal symbiosis also affected the metabolism of abscisic acid (ABA) during berry ripening [14]. Nevertheless, none of these studies detailed the profile of the main metabolites present in mature grapes collected from plants associated with AMF.

Therefore, the aim of the present study was to deepen on the effect of AMF on the profiles of primary and secondary metabolites determinants for the quality of fruits in Tempranillo grapevines.

## 2. Results and Discussion

### 2.1. Plant and Berry Traits

Mycorrhizal structures (mainly hyphae and vesicles) were observed in roots of inoculated grapevines, and the percentages of mycorrhizal colonization achieved values of around 30% (Table 1). No fungal structures were detected in roots of non-inoculated plants. However, there were no significant effects of mycorrhization on the plant growth (estimated as leaf area) neither on the bunch mass. Mycorrhizal association did not either significantly influence most of the berry traits determined in the study: berry mass, soluble solids, must pH, titratable acidity, color density, and tonality index (Table 1). However, it decreased the relative skin mass and increased the concentration of total phenolic compounds. Enhanced levels of total phenols in fruits have been found in different cultivars of strawberry when associated with AMF, but this effect is not universal. It depends on several factors: the host plant, the species and mixture of mycorrhizal fungi, the origin (native, commercial) of mycorrhizal inoculum, the time of mycorrhizal inoculation (early or late), the presence of other microorganisms accompanying AMF, and the nitrogen fertilization (NH_4_^+^, NO_3_^−^) among others [8]. In fact, the application of a commercial inoculum derived from an in vitro culture of *R. intraradices* did not induce the accumulation of total phenolic compounds in berries of Tempranillo CL-260 [12].

### 2.2. Anthocyanins and Flavonols in Berry Skin

Flavonoids are regarded as one of the most important determinants of quality in red grapes and wines. Color and taste of red wines are strongly related to the content of anthocyanins, flavonols, and proanthocyanidins. Moreover, in recent years some flavonoid compounds have attracted additional attention for their potential health benefits [15]. In order to enhance the levels of flavonoids (e.g., anthocyanins) in the edible parts of plants, AMF have been applied to several crops [8] because mycorrhizal inoculation can act as a biotic stress that may induce the expression of genes related to the synthesis of phenolic compounds [16]. In the present study, the content of total anthocyanins in berries from non-inoculated and inoculated grapevines was similar (Table 2). Although there have been cases in which levels of anthocyanins were increased in fruits of mycorrhized plants, results are dependent on several factors, such as the variety or cultivar of the host plant and the time of mycorrhizal inoculation [8]. Torres et al. [13] concluded that abiotic stresses (such as warming or drought) were more decisive than biotic factors (mycorrhization) for the accumulation of total anthocyanins in Tempranillo grapes. However, these previous studies did not provide information on the profile of anthocyanins present in the berries of Tempranillo grapevines inoculated with AMF. When the anthocyanins in berry skin were analyzed separately, we found that, regardless of mycorrhizal symbiosis, anthocyanins were dominated by malvidin-3-monoglucosides (Table 3), as observed for several other grapevine varieties [17,18]. The concentrations of delphinidin and petunidin in CL-260 non-inoculated with AMF were higher than those measured in other clones of Tempranillo [19], which reinforces previous findings showing intravarietal diversity within Tempranillo cultivar. These two anthocyanins (delphinidin and petunidin) improve the growth rate of *Saccharomyces cerevisiae*, the predominant yeast in winemaking [20]. Overall, our data show that mycorrhizal symbiosis exerted little influence on the profile of anthocyanins in berry skin, and only affected the levels of delphinidin-3-acetyl-glucoside decreased when plants were colonized by AMF (Table 3). In contrast with our results, Castellanos-Morales et al. [21] observed an increased concentration of cyanidin-3-glucoside in strawberry fruits when plants received an intermediate level of nitrogen fertilization.

Flavonols are a subclass of flavonoids considered to act as UV protectants and free-radical scavengers in berries [22,23]. Although they are colorless, flavonols are also thought to contribute to wine color as anthocyanin copigments [24]. As in the case of anthocyanins, we found no significant differences in total flavonols between non-inoculated and inoculated grapevines (Table 2), being the predominant flavonols myricetin followed by kaempferol (Table 3). However, flavonol profiling shows that mycorrhizal symbiosis increased the level of quercetin-3-*O*-galactoside and decreased that of quercetin-3-*O*-glucoside. Mollavali et al. [25] found enhanced levels of quercetin-4’-*O*-monoglucoside in bulbs of onion associated with *Diversispora versiformis*, but no effect was observed when plants were colonized by *Funneliformis mosseae* or *Rhizophagus intraradices*. This same study shows increased concentrations of isorhamnetin-4’-glucoside in bulbs of all mycorrhized onion plants. The amount of mycorrhizal inoculum applied to plants, the type of nitrogen form supplied, and the time elapsed from the AMF inoculation before the quantifications of flavonols occurred are key factors determining the efficiency of AMF in inducing the accumulation of flavonols in their host plants [25].

### 2.3. Sugars, Organic Acids and Amino Acids in Berry Skin

In general, our study shows that inoculation of Tempranillo with AMF modified more primary concentrations of berry skin than secondary metabolite concentrations (Table 2). Concerning primary metabolism, in previous work, Torres et al. [11] found similar levels of soluble sugars in leaves of mycorrhized and non-mycorrhized Tempranillo CL-260. In contrast, the concentrations of glucose in grapes were significantly higher in mycorrhized grapevines than in non-mycorrhized plants (Table 2). Different hypothesis can be posed to explain this apparent contradiction: (1) the quantification of total soluble sugars in leaves included some carbohydrates (for example, sucrose) not determined in fruits; (2) the transport of hexoses to grapes may be increased in plants associated with AMF. Zouari et al. [26] found that one of the most up-regulated genes in tomato fruits collected from mycorrhized plants was a hexose transporter with high sequence similarity to a glucose/H + symporter strongly induced at fruit maturity; (3) the mycorrhizal fungi colonizing roots of Tempranillo CL-260 may have exerted an important systemic effect on the fruit metabolism through a differential regulation of genes involved in carbohydrate metabolism [26]. As a consequence of this higher sugar content, grapes from mycorrhized plants could produce wines with greater alcoholic gradation after the fermentation process carried out by the yeasts. By contrast, the presence of mycorrhizal fungi in roots of Tempranillo did not affect the levels of malic or tartaric acids (Table 2). 

**Figure 1:**
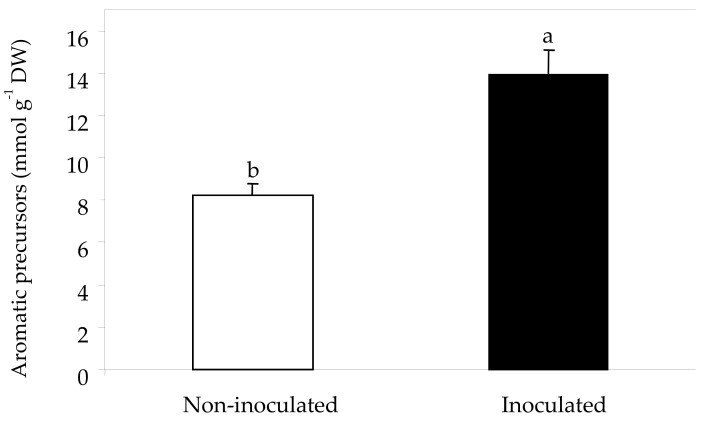
Concentration of aromatic precursors in berry skins of Tempranillo non-inoculated (**a**) or inoculated (**b**) with arbuscular mycorrhizal fungi. Values represent means (n=3). One-way ANOVA was performed to evaluate the effect of mycorrhizal inoculation. Means followed by different letter indicate that values are significantly different (P > 0.05). DW, dry weight.

Nitrogen is crucial for grapevine and winemaking because it affects the development of plants and yeasts, with consequent effects on wine quality [27]. In order to increase the levels of this element in grapevines, nitrogen compounds (urea or amino acids) have been applied to vineyard foliage or soil [27,28]. Although AMF have been widely studied in relation to an enhanced phosphorus status of their host plants, Scandellari [29] demonstrated that these edaphic fungi play an important role in the nitrogen nutrition of grapevines. Other studies carried out with different host plants have concluded that AMF favor the transport of some amino acids from colonized roots to the aerial part, including fruits. Tian et al. [30] suggested that the ammonium absorbed by the mycorrhizal hyphae is incorporated in the amino acid arginine prior being transferred from the extra-radical to the intra-radical mycelium and Whiteside et al. [31] concluded that mycorrhizal fungi can improve the uptake of some amino acids (arginine among them) by using colonized roots. In this same line, Salvioli et al. [32] hypothesized that the increased levels of glutamine and asparagine in tomato fruits of plants associated with AMF were a consequence of an enhanced production of these amides in mycorrhized roots followed by their transport from the source (roots) to the sink (fruits) organs. Zouari et al. [26] also noted that mycorrhizal symbiosis up-regulated genes related to amino acid synthesis in tomato fruits. Results obtained in our study showed that the total amount of amino acids (Table 2) was higher in berries from mycorrhized grapevines because the concentrations of serine, tyrosine, phenylalanine, aspartic acid, asparagine, threonine, isoleucine, arginine, aminobutyric acid, alanine, and valine were significantly increased in the skin of berries collected from grapevines associated with AMF in comparison with non-mycorrhizal plants (Table 4). This amelioration of total amino acid levels of berries may have a strong impact on the secondary aromas of the wine from mycorrhizal plants [33,34]. However, some of the free amino acids present in grapes can be transformed into biogenic amines during the wine making process as a consequence of their decarboxylation by microorganisms. This is the case for some of the amino acids whose levels were increased by the association of Tempranillo with AMF (Table 4): tyrosine is the precursor of tyramine, a strong vasoconstrictive amine [35]; phenylalanine is the precursor of phenylethylamine, although it is also a key compound for the synthesis of polyphenols; histidine is the precursor of histamine, which can cause food poisoning [35,36]; finally, arginine is the precursor of putrescine. According to Wang et al. [37], tyramine, phenylethylamine and putrescine are very common in musts and their levels account for around one-third of biogenic amines in wines. Thus, mycorrhization of Tempranillo induced the accumulation of threonine, closely correlated with the levels of odorants related to fatty acid synthesis (Table 2) [33]. The levels of isoleucine and valine were also higher in berries from grapevines colonized by AMF, which can favor the posterior accumulation of volatile compounds in wine [34]. Finally, Figure 1 clearly shows that berries from mycorrhizal plants achieved a higher concentration of all aromatic precursor amino acids (Asp, Ile, Phe, Thr, Tyr, and Val) than non-mycorrhizal plants. This fact could contribute to improving the aromatic flavor of the final wine [38].

## 3. Materials and Methods 

### 3.1. Biological Material and Growth Conditions

The present study was carried out with dormant cuttings of *Vitis vinifera* L. cv. Tempranillo, clone (CL) 260. Three-node segments 400-500 mm long were collected during the winter from an experimental vineyard of the Institute of Sciences of Vine and Wine (Logroño, Spain). The CL-260 comes from San Vicente de la Sonsierra (La Rioja, North of Spain, 42°33’44’’N 2°45’33’’O, 497 masl) and shows a short reproductive cycle in the field. 

Fruit-bearing cuttings were obtained according to the steps originally outlined by Mullins [39] and modified by Ollat et al. [40] and Antolín et al. [41]. Cuttings were rooted in a heat-bed (27°C) kept in a cool room (4°C). One month later, the cuttings were planted in 6.5 L plastic pots containing a mixture of vermiculite-sand-light peat (2.5:2.5:1, v:v:v). The peat (Floragard, Vilassar de Mar, Barcelona, Spain) had a pH of 5.2–6.0, 70–150 mg L^−1^ of N, 80–180 mg L^−1^ P_2_ O_5_, and 140–220 mg L^−1^ K_2_ O and was previously sterilized at 100°C for 1 h on three consecutive days. At transplanting, half of the plants (+M) were inoculated with the mycorrhizal inoculum Bioradis Gel (Bioera SLU, Tarragona, Spain). The inoculum was a mixture of five AMF (*Septoglomus deserticola*, *Funneliformis mosseae, Rhizoglomus intraradices, Rhizoglomus clarum,* and *Glomus aggregatum*), and contained 100 spores per g of inoculum and a mixture of rhizobacteria belonging to the genera *Bacillus* and *Paenibacillus* (2 × 10^6^ CFU g^−1^). Mycorrhizal inoculum was produced by using trap plants for each type of mycorrhizal fungus, and then all AMF were mixed according the commercial formulation. The final commercial formulation included plant growth promoting rhizobacteria (PGPRs) because they can act synergistically with AMF to benefit host plants (Hernández, A. from Bioera SLU, personal communication). The microbial preparation was diluted in distilled water (1:20) to ensure that each plant could receive 1 g of product. The inoculation was performed by submerging roots of fruit-bearing cuttings in the Bioradis Gel for 15 min. In order to restore rhizobacteria and other soil free-living microorganisms accompanying AMF, uninoculated plants (-M) were submerged for 15 min in a filtrate of the abovementioned mycorrhizal inoculum. The filtrate was obtained by passing mycorrhizal inoculum through a layer of 15–20 µm filter paper with particle retention of 2.5 µm (Whatman 42; GE Healthcare, Little Chalfont, UK). Microorganisms accompanying AMF play an important role in the uptake of soil resources as well as on the infectivity and efficiency of AMF isolates [42] and some PGPR, such as *Bacillus* spp. isolated from vineyards are known to benefit the basal immunity of grapevines against some pathogens [43]. By restoring the bacterial component of the mycorrhizal inoculum in the rhizosphere of the –M plants, differences between –M and +M plants are expected to be mainly due to the presence of AMF associated with +M plants. Then +M and –M plants were transferred to the greenhouses (six plants for each treatment). Growth conditions were 24/14°C (day/night), according to the average temperature registered in La Rioja during the growing season (1981-2010) [44], and 50/90% relative humidity (day/night) regime. Natural daylight (photosynthetic photon flux density, PPFD, was on average 850 μmol m^−2^ s^−1^ at midday) supplemented with high-pressure sodium lamps (SON-T Agro Phillips, Eindhoven, Netherlands) to extend the photoperiod up to 15 h and ensure a minimum PPFD of 350 μmol m^−2^ s^−1^. Humidity and temperature were controlled by using M22 W2 HT4 X transmitters (Rotronic Instrument Corp., Hauppauge, USA). PPFD was monitored with a LI-190 SZ quantum sensor (LI-COR, Lincoln, USA). In order to avoid the excessive soil warming, pots were wrapped with a reflecting material [45,46]. Soil temperature was measured at 5 cm soil depth using probes PT100 (Coreterm, Valencia, Spain) and reached 23 ± 0.5°C. Plants were watered twice per day (140 mL day^−1^) with the nutrient solution detailed by Ollat et al. [40]. The electric conductivity of the nutrient solution adjusted to pH 5.5 was 1.46 ± 0.15 mS cm^−1^ as determined with a conductivity meter 524 Crison (Crison Instruments S.A., Alella, Spain) and the phosphorus (P) level was 9.78 mg L^−1^. Plants were harvested and berry samples were collected at commercial maturity (22°Brix) (Eichhorn and Lorenz (E‒L) fruit stage 38) [47]. The time elapsed from the rooting of the cuttings to the ripening of the fruits was 93-95 days, both in -M and +M plants.

### 3.2. Determination of Plant and Berry Traits

#### 3.2.1. Plant Traits 

For determining mycorrhizal colonization, root samples were cleared and stained following the procedure described in Koske and Gemma [48]. A potassium hydroxide solution (10% w:v) was added to the roots which were placed in an oven at 70°C for 2 h. After rinsing with water, roots were clarified by the addition of H_2_ O_2_ (3% v:v) and subsequent washing with water. Then, they were acidified by soaking in HCl (1% v:v) for 5-15 min and stained in a solution of methyl blue: lactic acid (1% w:v) at 70°C for 1 h. Stained roots were stored in a mixture of glycerol, water, and HCl 1% (500:450:50, v:v:v) until quantification. The percentage of mycorrhizal colonization was determined under a stereoscopic microscope (15-20 x overall magnification) by the plate intersection method (100–130 intersections for each sample, one sample per plant) [49]. 

Total leaf area was measured with a portable area meter (model LI-3000, Li-Cor, Lincoln, Nebraska, USA). At commercial maturity (E‒L 38) bunches were weighed and then, ten berries from each plant were collected and weighed individually. The relative skin mass was calculated as the quotient between skin FW and total berry FW expressed as a percentage. The rest of berries were used to estimate the ratio between fresh dry weight (FW) and dry weight (DW) or frozen at -80 °C for further analysis. 

#### 3.2.2. Berry Traits 

A subsample of 25 berries was crushed and then extracts were centrifuged at 4300 *g* at 4 °C for 10 min. The supernatant was used for determination of total soluble solids (mainly sugars) measured with a temperature-compensating refractometer (Zuzi model 315; Auxilab, Beriain, Spain) and expressed as °Brix. Must pH was measured with a pH meter (Crison Instruments, Barcelona, Spain) standardized to pH 7.0 and 4.0; titratable acidity measured by titration with NaOH according to International Organisation of Vine and Wine methods [50], and expressed as g tartaric acid L^–1^.

Total phenolic compounds were determined in other subsamples of 25 mature berries (E‒L 38) per plant, which were ground to a powder in a ball grinder MM200 (Retsch, Haan, Germany). After adding 3 mL 80% aqueous acidified methanol (2% HCl 12 N) [51], phenolics were extracted by shaking samples overnight at room temperature in the dark. Then, samples were centrifuged at 13,200 g for 15 min at ambient temperature. The residues were re-extracted other two more times (for 3 h every re-extraction) under similar conditions. Supernatants were combined (9 mL in total for each sample) before determining phenolic compounds. Total phenolic compounds were spectrophotometrically determined as described by Lima et al. [52]. Samples (50 µL) were diluted (1:20) with 950 µL of aqueous ethanol (95% v:v) acidified with 0.1% HCl. Then other 4 mL of 2% HCl were added until a total final volume of 5 mL was obtained. The absorbance was measured at 280 nm and gallic acid was used as a standard. Absorbance was read in a UV-VIS spectrophotometer (UV 1800, Shimadzu, Tokyo) and results were expressed as mg of gallic acid per gram of berry DW.

Color density was calculated by adding the absorbance readings at 420, 520, and 620 nm, and the tonality index was determined by the ratio between the measured absorbance readings at 420 and 520 nm according to International Organisation of Vine and Wine methods [50]. 

### 3.3. Berry Skin Metabolites

#### 3.3.1. Anthocyanins and Flavonols 

**Figure 2:**
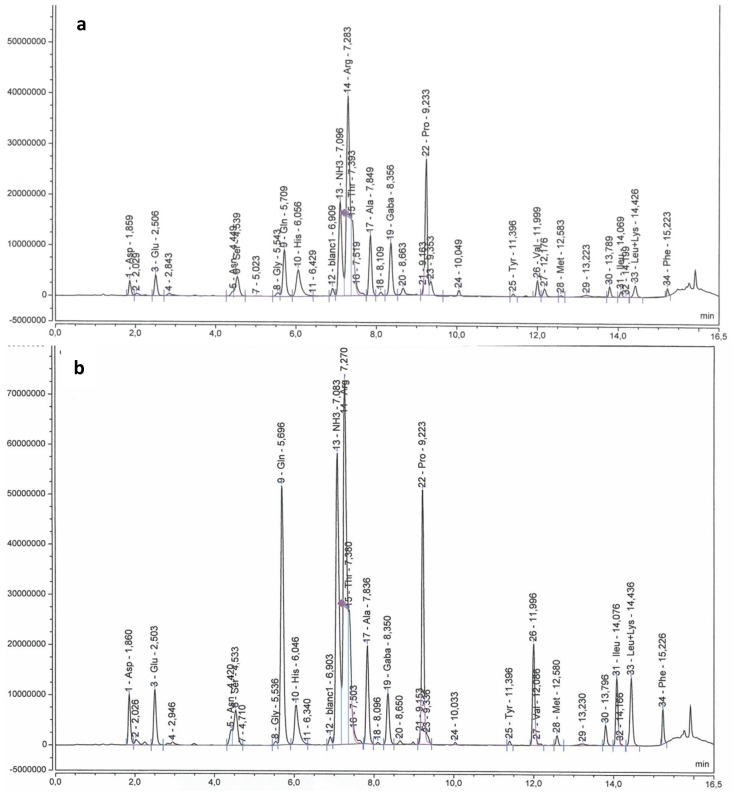
HPLC chromatograms of amino acid profile from berry skins of Tempranillo non-inoculated (**a**) or inoculated (**b**) with arbuscular mycorrhizal fungi. Asp: Aspartic acid; Glu: Glutamic acid; Asn: Asparagine; Ser: Serine; Gly: Glycine; Gln: Glutamine; His: Histidine; Arg: Arginine; Thr: Threonine; Ala: Alanine; GABA: γ-aminobutyric acid; Pro: Proline; Tyr: Tyrosine; Cys: Cysteine; Val: Valine; Met: Methionine; Ileu: Isoleucine; Lys: Lysine; Leu: Leucine; Phe: Phenylalanine. Excitation and emission wavelengths were 250 nm and 395 nm, respectively.

**Figure 3:**
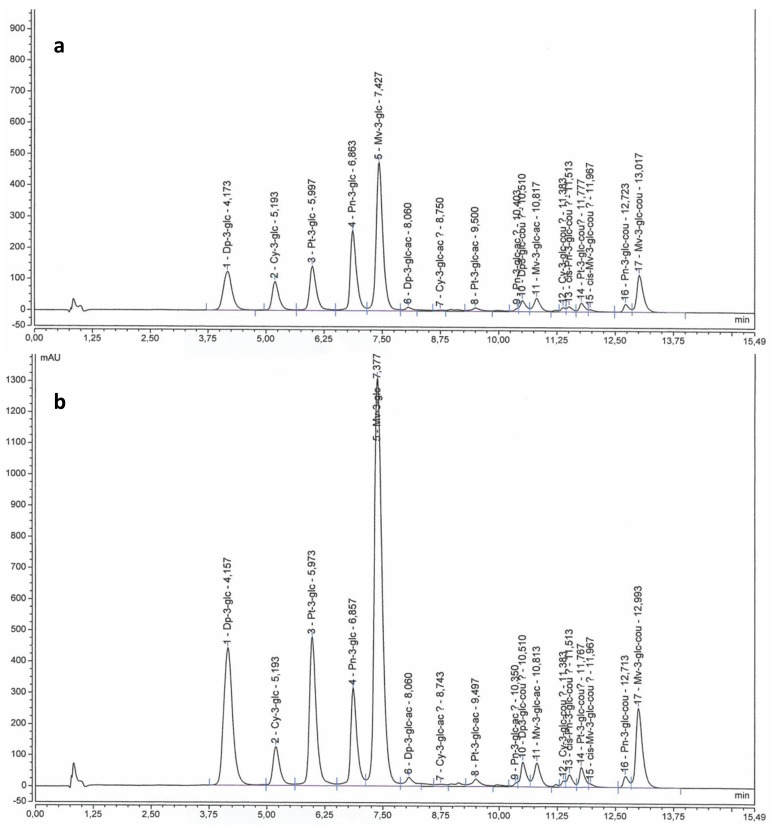
HPLC chromatograms showing the anthocyanin profile from berry skins of Tempranillo non-inoculated (**a**) or inoculated (**b**) with arbuscular mycorrhizal fungi. Dp-3-glc: Delphinidin-3-glucoside; Cy-3-glc: Cyanidin-3-glucoside; Pt-3-glc: Petunidin3-glucoside; Pn-3-glc: Peonidin-3-glucoside; Mv-3-glc: Malvidin-3-glucoside; Dp-3-glc-ac: Delphinidin-3-acetyl-glucoside; Cy-3-glc-ac: Cyanidin-3-acetyl-glucosides; Pt-3-glc-ac: Petunidin-3-acetyl-glucosides; Pn-3-glc-ac: Peonidin-3-acetyl-glucosides; Dp-3-glc-cou: Delphinidin-3 *p*-coumaroyl-glucoside; Mv-3-glc-ac: Malvidin-3-acetyl-glucosides; Cy-3-glc-cou: Cyanidin-3 *p*-coumaroyl-glucoside; *cis* Pn-3-glc-cou: *cis*-Peonidin-3 *p*-coumaroyl-glucoside; Pt-3-glc-cou: Petunidin-3 *p*-coumaroyl-glucoside; *cis*-Mv-3-glc-cou: *cis*-Malvidin-3 *p*-coumaroyl-glucoside; Pn-3-glc-cou: Peonidin-3 *p*-coumaroyl-glucoside; Mv-3-glc-cou: Malvidin-3 *p*-coumaroyl-glucoside. A detection wavelength of 520 nm was used.

Samples of 5-10 mature berries (E‒L 38) per plant were separated into skin and flesh (three plants for each treatment). Skins were powdered separately in a ball grinder MM200 (Retsch, Haan, Germany) and then, they were freeze dried in a lyophilizer Vir Tis Bench Top K (SP Scientific, Warminster, Philadelphia, USA). Skins from each plant were used to analyze primary and secondary metabolites. A subsample of powdered berry skins was used in order to analyze individual anthocyanins and flavonols. Samples were extracted according to Acevedo de la Cruz et al. [53] then analyzed as described in Martínez-Lüscher et al. [22] with some modifications. Extracts were analyzed using an UltiMate 3000 UHPLC system (Thermo Electron SAS, Waltham, MA USA) equipped with DAD-3000 diode array detector operating at 520 nm and at 360 nm (Thermo Electron SAS, Waltham, MA USA). Separation was performed on a Syncronis C18, 2.1 × 100 mm, 1.7 µm Column (Thermo Fisher Scientific, Waltham, MA USA) at 25°C with elution at 0.368 mL min^−1^ according to the following gradient (v/v): 0 min 92.2% A 7.8% B, 9.6 min 73% A 27% B, 14.1 min 70% A 30% B, 14.8 min 92.2% A 7.8% B (eluent A, water and formic acid, 90/10 v/v; eluent B, acetonitrile). Identification and peak assignment of phenolic compounds were based on comparison of their retention times and UV-Vis spectrometric data with that of pure standards. Formal identification of flavonoids was performed by liquid chromatography coupled to mass spectrometry and nuclear magnetic resonance spectrometry in previous works [53,54]. Chromeleon software, version 7.1 (Thermo Electron SAS, Waltham, MA US-States) was used to calculate peak area. The concentration of individual flavonoids was calculated in milligrams per gram (mg g^−1^) of skin DW using malvidin-3-*O-*glucoside was used as external standard for all the quantified anthocyanins (at 520 nm), and quercetin-3-*O*-glucoside was used for all the quantified flavonols (at 360 nm) (Extrasynthese, Genay, France). Chromatographic profiles of anthocyanins and flavonoids are shown, respectively, in Figure 2 and Figure 3.

#### 3.3.2. Sugars, Organic Acids and Amino Acids 

Another subsample of powdered berry skins was used in order to analyze sugars, organic acids and individual amino acids (three plants for each treatment). Primary metabolites were extracted according to Bobeica et al. [55] with minor modifications. Subsamples of 50 mg fine powder of skins were extracted with 80% ethanol (v/v) at 80°C for 15 min followed by two extractions with 50% ethanol (v/v) and ultrapure water respectively, dried in Speed-Vac, and re-dissolved in ultrapure water. Resultant extracts were used for determinations of sugars, organic acids and amino acids. 

Sugars were measured enzymatically with an automated micro-plate reader (Elx800 UV, Biotek Instruments Inc., Winooski, Vermont, USA) using the Glucose/Fructose kit from BioSenTec (Toulouse, France). Malic acid was determined using an enzyme-coupled spectrophotometric method that measures the change in absorbance at 340 nm from the reduction of NAD^+^ to NADH. Tartaric acid was assessed by using the colorimetric method based on ammonium vanadate reactions [56]. Both compounds were quantified with a Bran and Luebbe TRAACS 800 autoanalyzer (Bran & Luebbe, Plaisir, France). 

**Figure 4:**
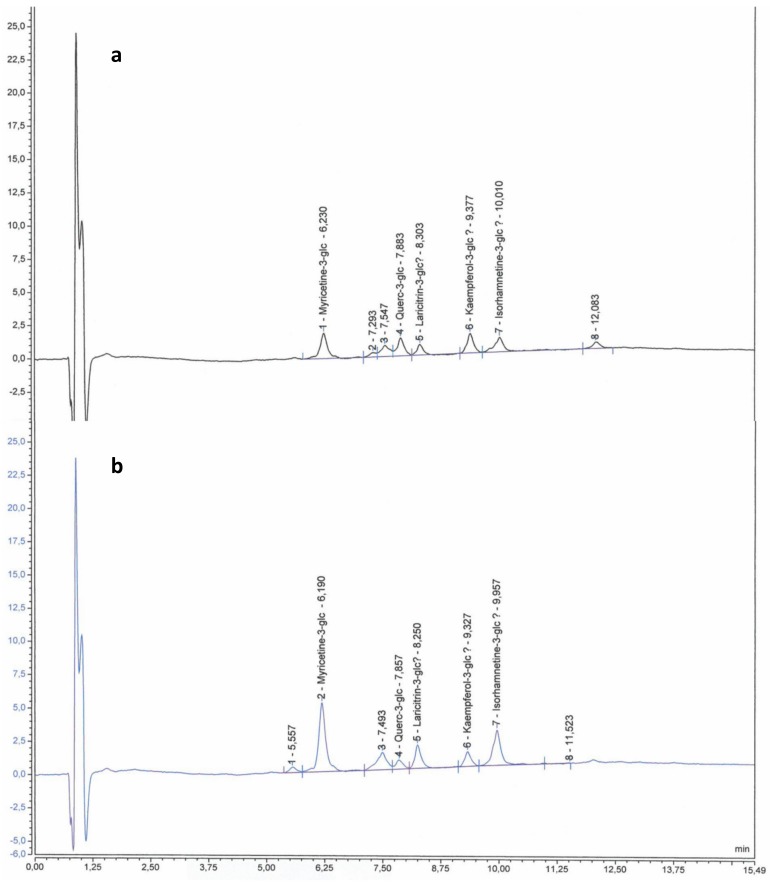
HPLC chromatograms showing the flavonol profile from berry skins of Tempranillo non-inoculated (**a**) or inoculated (**b**) with arbuscular mycorrhizal fungi. Myricetin-3-glc: Myricetin-3-*O*-glucoside; Querc-3-glc: Quercetin-3-*O*-glucoside; Laricitrin-3-glc: Laricitrin-3-*O*-glucoside; Kaempferol-3-glc: Kaempferol-3-*O*-glucoside; Isorhamnetin-3-glc: Isorhamnetin-3-*O*-glucoside. A detection wavelength of 360 nm was used.

After derivation with 6-aminoquinolyl-N-hydroxy-succinimidyl-carbamate (AccQ-Tag derivatization reagent, Waters, Milford, USA) according to Hilbert et al. [57], free amino acids were measured according to Habran et al. [58]. Briefly, amino acids were analyzed using an UltiMate 3000 UHPLC system (Thermo Electron SAS, Waltham, USA) equipped with FLD-3000 Fluorescence Detector (Thermo Electron SAS, Waltham, USA). Separation was performed on a AccQ•Tag Ultra column, 2.1 × 100 mm, 1.7 μm (Waters, Milford, USA) at 37°C with elution at 0.5 mL min^−1^ (eluent A, sodium acetate buffer, 140 mM at pH 5.7; eluent B, acetonitrile; eluent C, water) according to the gradient described by Habran et al. [58]. Chromatograms corresponding to excitation at 250 nm and emission at 395 nm were recorded (Figure 4). To maintain consistent retention time and a stable baseline, a control was performed before each run of 18 samples to detect possible trouble in the chromatogram. Chromeleon software, version 7.1 (Thermo Electron SAS, Waltham, USA) was used to calculate the peak area. A standard of 20 amino acids (Alanine, Arginine, Aspartic acid, Asparagine, Cysteine, GABA, Glycine, Glutamic acid, Glutamine, Histidine, Isoleucine, Leucine, Lysine, Methionine, Phenylalanine, Proline, Serine, Threonine, Tyrosine, and Valine) purchased from Sigma (St Louis, Missouri, USA) was used after the control and in the middle of each run to calibrate amino acid quantification. Seventeen amino acids were identified and quantified in skin extracts as described by Pereira et al. [56]. Results were expressed in mmol g^−1^ skin DW. The concentration of aromatic precursors was calculated by summing the Asp, Ile, Phe, Thr, Tyr, and Val concentrations [38].

### 3.4. Statistical Analysis

Statistical analyses were carried out using statistical software the Statistical Package for the Social Sciences (SPSS) (SPSS Inc., Chicago, IL, USA) version 22.0 for Windows. All data were subjected to a one-way analysis of variance (ANOVA) after establishing the normality of the data with the Kolmogorov-Smirnov normality test due to the small sample size (n= 3-6). Means ± standard errors (SE) were calculated and when the F ratio was significant (P≤0.05), a Duncan test was applied. 

## 4. Conclusions

Mycorrhizal symbiosis modified the profile of metabolites in berries of Tempranillo CL-260. The strongest effect was exerted on glucose and amino acids, whose levels significantly increased in berries of mycorrhized grapevines, including those of the aromatic precursor amino acids. In contrast, AMF barely influenced the amount and the profiles of phenolic compounds. Our results suggest that mycorrhizal inoculation of grapevines may be an alternative to the exogenous application of nitrogen compounds in order to enhance the contents of amino acids in grapes, which may affect the aromatic characteristics of wines.

## Figures and Tables

**Table 1:** Plant and berry characteristics from fruit-bearing cuttings of Tempranillo inoculated or non-inoculated with arbuscular mycorrhizal fungi.

	Non-Inoculated	Inoculated
Plant traits		
Mycorrhization (%)	-	31.5
Leaf area (m^2^ plant^−1^)	0.79 a	0.84 a
Bunch mass (g plant^−1^)	182.3 a	198.5 a
Berry traits		
Berry mass (g berry^−1^)	1.24 a	1.22 a
Relative skin mass (% berry FW)	33.3 a	23.9 b
Total soluble solids (°Brix)	22.8 a	21.9 a
Must pH	3.7 a	3.6 a
Titratable acidity (g L^−1^)	7.11 a	6.94 a
Total phenolic compounds (mg g^−1^ DW)	62.24 b	84.17 a
Color density (AU)	20.9 a	20.4 a
Tonality index	0.53 a	0.52 a

Values represent means (n = 6). One-way ANOVA was performed to evaluate the effect of mycorrhizal inoculation. Within each file, means followed by the same letter indicate that values are not significantly different (P ≥ 0.05). AU, absorbance units. FW, fresh weight.

**Table 2:** Main metabolite groups measured at harvest in grapes of fruit-bearing cuttings of Tempranillo inoculated or non-inoculated with arbuscular mycorrhizal fungi.

	Non-Inoculated	Inoculated
**Secondary metabolites**		
Anthocyanins (mg g^−1^ DW)	36.3 a	34.8 a
Flavonols (mg g^−1^ DW)	1.01 a	0.96 a
**Primary metabolites**		
Glucose (mg g^−1^ DW)	98.9 b	147.3 a
Fructose (mg g^−1^ DW)	100.1 a	129.5 a
Malic acid (mg g^−1^ DW)	19.9 a	28.4 a
Tartaric acid (mg g^−1^ DW)	22.8 a	29.4 a
Total amino acids (mmol g^−1^ DW)	89.4 b	129.3 a

Values represent means (n = 3). One-way ANOVA was performed to evaluate the effect of mycorrhizal inoculation. Within each file, means followed by the same letter indicate that values are not significantly different (P ≥ 0.05). DW, dry weight.

**Table 3:** Anthocyanin and flavonol profiles measured at harvest in grape skins of fruit-bearing cuttings of Tempranillo inoculated or non-inoculated with arbuscular mycorrhizal fungi.

	Concentration (mg g^−1^ DW)	
	Non-Inoculated	Inoculated
**Anthocyanins**		
3-Monoglucosides		
Delphinidin	8.79 a	7.90 a
Cyanidin	3.62 a	2.99 a
Petunidin	5.88 a	5.43 a
Peonidin	4.42 a	4.82 a
Malvidin	11.16 a	11.02 a
3-Acetyl-glucosides		
Delphinidin	0.36 a	0.26 b
Petunidin	0.25 a	0.19 a
Malvidin	0.37 a	0.36 a
3 p-Coumaroyl-glucosides		
Peonidin	0.28 a	0.38 a
Malvidin	1.49 a	1.39 a
**Flavonols**		
Myricetin-3-O-glucoside	0.43 a	0.48 a
Quercetin-3-O-galactoside	0.02 b	0.04 a
Quercetin-3-O-glucoside	0.13 a	0.08 b
Laricitrin-3-O-glucoside	0.05 a	0.06 a
Kaempferol-3-O-glucoside	0.22 a	0.22 a
Isorhamnetin-3-O-glucoside	0.09 a	0.08 a

Values represent means (n = 3). One-way ANOVA was performed to evaluate the effect of mycorrhizal inoculation. Within each file, means followed by the same letter indicate that values are not significantly different (P ≥ 0.05). DW, dry weight.

**Table 4:** Amino acid profiles measured at harvest in grape skins of fruit-bearing cuttings of Tempranillo inoculated or non-inoculated with arbuscular mycorrhizal fungi.

Precursor	Amino Acid	Concentration (mmol g^−1^ DW)	
		Non-Inoculated	Inoculated
3-Phosphoglycerate	Glycine	0.31 a	0.26 a
	Serine	1.89 b	2.91 a
Phosphoenolpyruvate	Tyrosine	0.21 b	0.49 a
	Phenylalanine	0.29 b	0.57 a
Oxaloacetate	Aspartic acid	2.33 a	3.86 a
	Asparagine	2.35 b	5.03 a
	Threonine	4.65 b	7.49 a
	Methionine	0.01 a	0.01 a
	Isoleucine	0.20 b	0.42 a
α-ketoglutarate	Glutamic acid	2.62 b	5.53 a
	Glutamine	6.84 a	8.16 a
	Histidine	0.91 a	0.99 a
	Arginine	25.97 b	39.65 a
	γ-aminobutyric acid	3.40 b	5.10 a
	Proline	38.35 a	43.53 a
Pyruvate	Alanine	3.88 b	6.83 a
	Valine	0.54 b	1.12 a

Values represent means (n = 3). One-way ANOVA was performed to evaluate the effect of mycorrhizal inoculation. Within each file, means followed by the same letter indicate that values are not significantly different (P ≥ 0.05). DW, dry weight.
